# The effects of acid-sensing ion channel-1A on conditioned fear memory are age-dependent

**DOI:** 10.1016/j.ibneur.2026.08.002

**Published:** 2026-08-05

**Authors:** R.J. Taugher-Hebl, A. Berns, M. Jones, A. Townsend, A. Eagen, Langbehn, H. Janouschek

**Affiliations:** a Department of Psychiatry, Iowa Neuroscience Institute, Carver College of Medicine, University of Iowa, Iowa City, IA, USA; b Departments of Psychiatry and Biostatistics, Carver College of Medicine and Iowa College of Public Health, University of Iowa, Iowa City, IA, USA; c Department of Veterans Affairs Medical Center, Iowa City, IA, USA

**Keywords:** ASIC1A, Acid sensing ion channel-1A, Fear, Fear conditioning, Development, Memory

## Abstract

The neuronal circuits underlying Pavlovian fear conditioning have been suggested to undergo marked changes during brain development. One molecule that is expressed in the brain during early development and that plays a key role in fear learning and fear memory in adult mice is the acid-sensing ion channel-1A (ASIC1A). Therefore, this gene is well-positioned to influence Pavlovian fear conditioning across development. To explore this possibility, we used Pavlovian fear conditioning to probe the effect of *Asic1a* disruption on fear memory acquisition and on cued- and contextual fear memory recall throughout development. During fear memory acquisition, *Asic1a*^*-/-*^ mice of all ages froze significantly less than *Asic1a*^*+/+*^ mice. Cue-evoked freezing tested 24 h after training was similarly reduced in *Asic1a*^*-/-*^ mice trained at postnatal day 23 (P23) and later. Surprisingly, cue-evoked freezing was unimpaired in *Asic1a*^*-/-*^ mice trained at P17. However, context evoked freezing was largely impaired across development, implying cue and context freezing are dissociable at P17. To our knowledge this is the first report of age-dependent behavioral effects with loss of ASIC1A. The emergence of cue-evoked memory deficits in *Asic1a*^*-/-*^ mice between P17 and P23 coincides with the extensive developmental alterations the fear circuit undergoes during this time window. The increasing deficit in fear memory with age in *Asic1a*^*-/-*^ mice further suggests a role for ASIC1A in the maturation or function of circuits relevant to Pavlovian fear conditioning.

## 1 Introduction

Fear learning and cue- and context-evoked fear-responses are essential to assess potentially deadly situations and escape potential predators based on prior experience. This capacity becomes vital when a young animal becomes increasingly independent from its mother and starts to explore the world on its own, which corresponds to the late pre-weaning and early weaning period. To facilitate the development of those crucial skills, the fear circuit undergoes significant changes during brain development, and as a result fear processing in early life and adulthood differ substantially (Meyer and Lee, 2019, Tallot et al., 2016). Therefore, genes known to impact fear learning and/or processing in adulthood might have different effects during earlier stages of development.

Performing Pavlovian fear conditioning at different developmental stages provides a way to probe effects across development. Pavlovian fear conditioning is a well-established model to assess fear memory with age-dependent conditioned responses (Akers et al., 2012, Akers et al., 2014, Pattwell et al., 2011, Taugher-Hebl et al., 2025). Contextual fear learning begins to emerge at P15, though it is not stably evoked 24 h after training until P17 (Akers et al. 2012). This contextual fear memory acquired at P17 is reduced to about 50% 1 week after training (Akers et al. 2014). However, the expression of contextual fear memory undergoes further developmental fluctuations. For example, juvenile mice (P29 - 33) have been reported to show hardly any context-evoked freezing but do exhibit significant cue-evoked freezing at 24–48 h after training (Pattwell et al. 2011). These developmentally regulated behavioral changes are accompanied by a significant structural and functional maturation of the fear circuit and its key structure, the basolateral amygdala (BLA), a region essential for fear memory acquisition and retrieval. In this region two types of neurons essential for Pavlovian fear conditioning are principal neurons and parvalbumin-positive interneurons (Yau et al., 2021, Wolff et al., 2014, Krabbe et al., 2018). While principal neurons are critical for plasticity during fear memory acquisition, parvalbumin-positive interneurons are essential for fear memory acquisition and expression. Within the first postnatal month, both neuron populations undergo significant maturation. On a morphological level, these developmental changes include increasing spine densities in principal neurons (Berdel and Moryś, 2000, Ryan et al., 2016). On a molecular level, these changes include increasing parvalbumin expression in parvalbumin-positive interneurons, alterations in NMDA and glutamate receptor subunit expression, and changes in protein phosphorylation (e.g., of CREB, GluA1). These developmental changes raise questions about how genes that affect fear memory in adulthood impact fear across development. There are several possibilities: 1) a uniform effect across ages, 2) a genetic effect that increases with ongoing development 3) and a genetic effect that is only present after maturation, in adulthood. Moreover, effects on cued and context could track together or could dissociate. To probe these possibilities, it is necessary to study genes relevant to adult fear learning and fear memory at different developmental timepoints.

One molecule that is well-positioned to play a developmental role in fear learning and fear memory is the acid-sensing ion channel−1A (ASIC1A). ASIC1A is a cation channel that is activated by extracellular acidosis (Storozhuk et al., 2021, Zhu et al., 2022, Wemmie et al., 2013, Zha, 2013). It is localized to dendritic spines, where it has been implicated in dendritic spine dynamics, synaptic plasticity and neurotransmission (Zha et al., 2006, Kreple et al., 2014; Du et al., 2014; Liu et al. 2016; Chiang et al. 2015; Wemmie et al. 2002; González-Inchauspe et al. 2017). In adult mice, ASIC1A is expressed in multiple regions of the fear circuit, including the BLA, where it is present in interneurons and principal cells and where it plays a key role in long-term potentiation (Wemmie et al., 2003, Du et al., 2017, Du et al., 2014, Chiang et al., 2015, Taugher et al., 2017, Pidoplichko et al., 2014, Wemmie et al., 2002). Adult mice that lack ASIC1A (*Asic1a*^*–/–*^ mice) have deficits in a variety of fear-related behaviors such as cued and context fear conditioning, TMT-evoked freezing and avoidance, CO_2_-evoked freezing and avoidance, acoustic startle, and center avoidance in the open field (Wemmie et al., 2003, Coryell et al., 2007, Ziemann et al., 2009, Taugher et al., 2014, Price et al., 2014). Though most studies on the role of ASIC1A have used adult mice, it has been reported that ASIC1A is expressed in the brain as early as embryonic day 12 (Alvarez de la Rosa et al. 2003), raising the possibility that it may play important roles throughout development.

Therefore, we hypothesized that the effects of ASIC1A on fear memory acquisition and recall might differ in young vs. adult mice. To test this hypothesis, we conditioned separate groups of mice, ranging from the pre-weaning period (P17) to adulthood (P83). Specifically, we tested fear memory acquisition, cue-evoked fear memory 24 h and 15 days after training and context-evoked fear memory 48 h and 16 days after training.

## 2 Methods

### 2.1 Animals

**Fig. 1:**
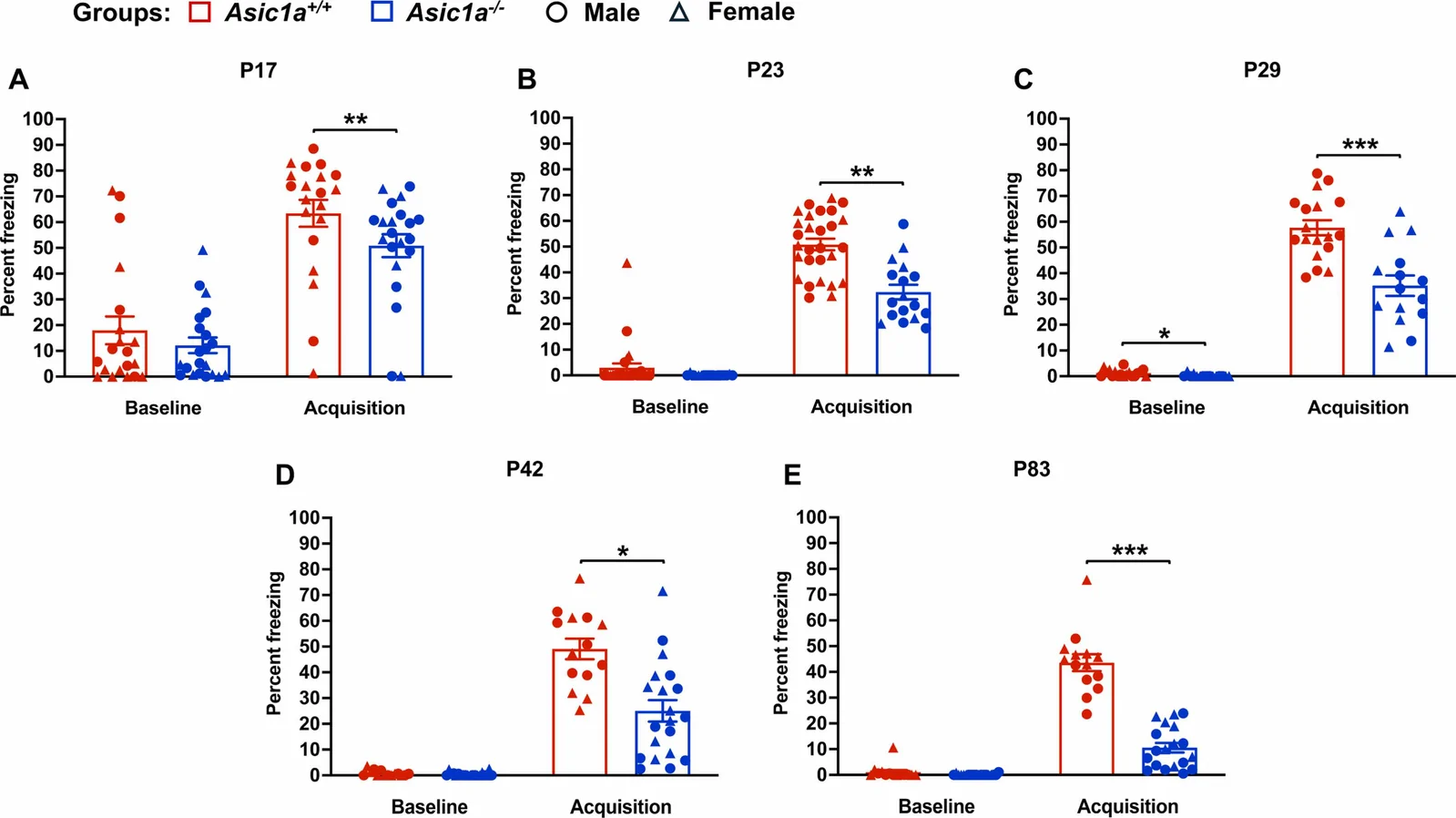
ASIC1A disruption impairs fear memory acquisition at all ages. Freezing at baseline and during fear memory acquisition in *Asic1a* knockout (^-/-^, blue) vs. wild-type (^+/+^, red) mice trained at: P17 (A); P23 (B); P29 (C); P42 (D); and P83 (E). N for P17: 20 wild-type (8 m, 12 f) and 21 knockout (13 m, 8 f) mice. N for P 23: 28 wild-type (14 m, 14 f) and 17 knockout (11 m, 6 f) mice. N for P29: 18 wild-type (10 m, 8 f) and 15 knockout (7 m, 8 f) mice. N for P42: 14 wild-type (7 m, 7 f) and 20 knockout (10 m, 10 f) mice. N for P83: 14 wild-type (7 m, 7 f) and 19 knockout (11 m, 8 f) mice. Baseline p-value in A-E) is based on square root transformation. *p < 0.05, **p < 0.005, ***p < 0.001.

*Asic1a*^*–/–*^ mice, were generated as previously described (Wemmie et al. 2002). Mice were maintained on a congenic C57BL/6 J background. Mouse ages at the start of the fear-conditioning experiment ranged from P17 to P83. The developmental stages tested were pre-weaning (P17), early post-weaning (P23), juvenile and adolescent (P29, P42) and adult (P83). For pre-weaning fear-conditioning experiments, littermate controls (*Asic1a*^*+/+*^ and *Asic1a*^*-/-*^) were generated by *Asic1a*^*+/-*^ x *Asic1a*^*+/-*^ breeding. For post-weaning fear conditioning experiments mice were generated by *Asic1a*^*–/–*^ x *Asic1*^*–/–*^ *and Asic1a*^*+/+*^ x *Asic1a*^*+/+*^ breeding. Mice were harem-bred, and dams were separated before giving birth. Distinct sets of mice were used for each age cohort. All mice were included in all experimental phases of their respective age groups. Numbers of mice and litters used can be found in the legend of Fig. 1 and in Supplementary Table 1. Mice of both sexes were used for experiments, and as detailed in the statistical analysis section, we controlled for potential sex effects and tested for potential sex-by-genotype interactions. Mice were given ad libitum access to standard chow (7913, Teklad Standard Irradiated Diet, Inotiv Teklad Madison, WI, USA) and water. They were kept at a 12-hour light-dark cycle in a temperature- and humidity-controlled facility. Weaning took place between P21 and P25. Animal care met the standards set by the National Institutes of Health and all experiments were approved by the University of Iowa Animal Care and Use Committee.

### 2.2 Fear conditioning

Fear conditioning took place in Med Associates (Fairfax, VT) fear conditioning chambers. Motion thresholds of the VideoFreeze™ software (Med Associates) were adjusted to mouse age based on hand scoring of a representative group of mice (see Supplementary Table 2). We used automated scoring by the Video Freeze software (Med Associates) to assess freezing and maximum motion during the first foot shock (shock reactivity).

All experiments took place during the light cycle. Mice were given at least 30 min of habituation in their home cage after transfer to the experimental area. At postnatal day (P) 17, 23, 29, 42 and 83, we conditioned separate groups of mice. For visualization of the timeline, see Supplementary Fig. 1.

Mice were trained (context A) on day 0 (D0). 1% bleach was used as contextual odor. Pre-weaning mice were habituated to the chamber for 5 min in the darkness, followed by 3 min in the light and post-weaning mice were habituated in the light for 3 min. The additional habituation time in pre-weaning mice was based on initial experiments (data not shown) which demonstrated non-specific freezing without those additional 5 min. Freezing during the habituation period in the light is referred to as baseline freezing in the analysis and discussion. Following habituation, a series of five tones (20 s, 90 db, 3000 Hz), co-terminating with a 1-second foot shock (0.75 mA) were presented. The inter-trial interval between tones was 120 s. The last tone presentation was followed by 80 s without tone or shock. The period from the onset of the first tone through the end of the last 80 s post-shock interval (i.e., the one after the fifth tone-shock) is referred to as acquisition or fear memory acquisition in the analysis and discussion. Cued fear memory recall was probed on day 1 (D1, 24 h after training) in a novel context (context B) with altered lighting, floor texture and odor (peppermint). After habituation (5 min in the dark and 3 min with light for pre-weaning mice and 3 min with light for post-weaning mice), a 3-minute 90 db, 3000 Hz tone was played, followed by additional 4 min without tones or foot shocks. Freezing while the tone (cue) was presented is referred to as freezing during cue presentation or cue-evoked freezing and corresponds to cue-evoked fear memory. Cued fear memory maintenance (context B) was tested on day 15 (D15) with the same protocol as used on day 1. Contextual fear memory recall was tested on day 2 (D2, 48 h after training) by 5-minute exposure to the training context (context A), regardless of age at training, because initial experiments with pre-weaning mice (data not shown) suggested that the duration of context exposure did not affect the extent of context freezing. On day 16 (D16), contextual fear memory maintenance was tested with the same context (context A) and protocol used on day 2.

### 2.3 Statistical analysis

To probe for genotype-specific behavioral differences within the same age group and for developmental effects on behavior, we used mixed effects linear regression analysis. In all mixed-effects models, we controlled for potential sibling correlations by including a random effect for litter. We controlled for potential sex effects by including fixed covariate effects for sex and genotype-by-sex interactions. Data are presented as mean ± standard deviations of the raw data. The reported p values are provided by the regression model. In the case of highly skewed distributions – as determined by our statistician, Dr. Langbehn – group comparisons were performed using the square root of the scores. Follow-up models tested for significant genotype differences in behavioral trends over time. As above, mixed-effect models with random effects were used. The fixed effect predictors were genotype, test day, their interaction (the main parameter of interest), and sex.

Given that there was little numerical difference between baseline freezing at day zero (training day) and freezing upon re-exposure to the training context at 48 h after training we performed follow up-analysis to probe if mice of each age group, genotype and sex retained memory about the training context. The variance within groups tended to be smaller at baseline testing than on follow-up. This was especially notable for mouse ages P29 and beyond. To account for this as well as for within-mouse and within-litter associations, we fit linear models that combined random effects per mouse and within-litter variance and correlation that varied by testing day. The test-day-specific within-litter effects were estimated by iterative weighted least squares. The model fixed effects saturated the possible combinations of genotype, sex, and test day, and the learning comparisons of interest were estimated by the corresponding linear contrasts of those effects. Statistical analysis was performed using R, version 4, using the libraries lmerTest (v3.1), lme4 (v1.1) and emmeans (v1.1) and SAS (Version 9.4 with STAT version 15.3) Proc Mixed. For all statistical analysis a p-value < 0.050 was considered statistically significant and a p-value < 0.100 was regarded as statistical trend. Graphs represent raw data. The error bars in the graphs represent standard errors of the means of the raw data.

## 3 Results

### 3.1 Baseline freezing is largely normal in *Asic1a*^*-/-*^ mice across development

*Asic1a*^*-/-*^ and *Asic1a*^*+/+*^ mice trained at P17, P23, or P42 showed similar baseline freezing on training day (P17: t(28.8) = 1.011, p = 0.320; P23: t(8.9) = 1.594, p = 0.146; P42: t(3.5) = 1.191, p = 0.309) (Fig. 1A, B, D; Table 1). Contrasting this, *Asic1a*^*-/-*^ mice trained at P29 showed reduced baseline freezing compared to *Asic1a*^*+/+*^ mice (t(7.1) = 2.400, p = 0.047) (Fig. 1C; Table 1). In mice trained at P83 we saw a trend towards lower baseline freezing for *Asic1a*^*-/-*^ mice (t(30.0) = 1.769, p = 0.087) (Fig. 1E; Table 1). Given the small numerical differences in baseline freezing, the biological significance of those numerical differences in baseline freezing remains unclear. We saw no significant sex-effects nor significant sex-by-genotype interactions at any age. The model which probed for effects of age on baseline freezing did not yield significant results (t(104.6) = −0.985, p = 0.327) and there was no age-by-genotype interaction (t(65.8) = −0.622, p = 0.536).

**Table 1:** Age-dependent effects of ASIC1A disruption on acquisition, recall and retention of conditioned fear memory.

**Age at training**	**Training baseline freezing**			**Training from 1**^**st**^**cue onset**				**Cued fear memory test 24 hrs. after training**				**Cued fear memory test 15 days after training**				**Contextual fear memory test 48 hrs. after training**				**Contextual fear memory test 16 days after training**			
	***Asic1a***^***+/+***^	***Asic1a***^***-/-***^	**p**		***Asic1a***^***+/+***^	***Asic1a***^***-/-***^	**p**		***Asic1a***^***+/+***^	***Asic1a***^***-/-***^	**p**		***Asic1a***^***+/+***^	***Asic1a***^***-/-***^	**p**		***Asic1a***^***+/+***^	***Asic1a***^***-/-***^	**p**		***Asic1a***^***+/+***^	***Asic1a***^***-/-***^	**p**
**P17**	18.0 ± 24.1	12.2 ± 13.9	0.320^**†**^		63.4 ± 23.4	50.9 ± 20.4	0.004*		58.1 ± 36.6	58.8 ± 35.7	0.922		29.3 ± 33.1	13.8 ± 21.9	0.143		43.6 ± 31.4	18.4 ± 24.3	0.009*^†^		14.5 ± 14.0	6.5 ± 8.8	0.036*^†^
**P23**	3.0 ± 8.8	0.1 ± 0.3	0.146^**†**^		50.8 ± 12.0	32.4 ± 11.7	0.001*		75.6 ± 30.3	51.7 ± 31.4	0.019*		75.5 ± 23.6	34.4 ± 22.1	< 0.001*		30.3 ± 22.3	7.9 ± 12.8	0.001*^†^		16.8 ± 12.6	6.7 ± 6.1	0.003*^†^
**P29**	1.3 ± 1.4	0.2 ± 0.6	0.047*^†^		57.7 ± 12.3	35.2 ± 15.4	< 0.001*		86.0 ± 19.6	46.3 ± 23.1	0.003*		70.5 ± 27.3	36.6 ± 26.3	0.021*		43.0 ± 23.2	21.7 ± 17.8	0.002*^†^		21.2 ± 26.0	8.3 ± 13.0	0.132^**†**^
**P42**	0.8 ± 0.1.1	0.4 ± 0.8	0.309^**†**^		49.1 ± 15.0	25.0 ± 18.6	0.014*		68.8 ± 25.5	29.7 ± 22.3	0.007		M: 81.7 ± 12.3 F: 72.4 ± 20.8	M: 15.5 ± 14.1 F: 39.1 ± 18.1	M: < 0.001* F: 0.005*		M: 37.4 ± 14.0 F: 19.8 ± 11.6	M: 5.8 ± 4.6 F: 12.3 ± 14.1	M: < 0.001*^†^ F: 0.144^**†**^		20.2 ± 11.1	6.4 ± 10.7	0.051^**†**^
**P83**	1.1 ± 2.8	0.1 ± 0.3	0.087^**†**^		43.6 ± 12.2	10.6 ± 8.1	< 0.001*		73.9 ± 14.6	15.1 ± 15.5	< 0.001*		69.6 ± 16.3	11.2 ± 9.7	< 0.001*		M: 16.2 ± 12.7 F: 42.6 ± 17.0	M: 11.5 ± 21.0 F: 7.7 ± 11.7	M: 0.318^**†**^ F: 0.009^†^*		18.8 ± 10.8	2.7 ± 5.1^**†**^	< 0.001*^†^

Table 1: Comparison of freezing between *Asic1a*^+/+^*and Asic1a*^-/-^ mice across development. In cases of significant genotype-by-sex interaction data for males and females are listed separately, in all other cases data for both sexes is combined. Data are presented as means ± standard deviation from the raw data. P values are calculated by the mixed effect linear effect model controlling for sex as an additional fixed effect and litter as a random effect. *Statistically significant within the fitted linear model. ^**†**^ Based on square root transformations for improved statistical significance accuracy.

### 3.2 The effect of ASIC1A disruption on fear memory acquisition increases with age

*Asic1a*^*-/-*^ mice trained at all ages showed lower freezing during fear memory acquisition than *Asic1a*^*+/+*^ mice (P17: t = (27.6)= 3.161, p = 0.004; P23: t(8.6) = 4.580, p = 0.001; P29: t(30.0) = 4.594, p < 0.001; P42: t(5.9) = 3.428, p = 0.014; P83: t(30.0) = 10.350, p < 0.001) (Fig. 1A-E, Table 1). A sex effect was observed for mice trained at P83, females freezing more than males (t(30.0) = −3.209, p = 0.003), but there was no genotype-by-sex interaction, so data are not separated by sex. In both genotypes, freezing during fear memory acquisition tended to decrease with age, however this decrease was only significant in *Asic1a*^*-/-*^ mice (*Asic1a*^*-/-*^: t(65.7) = −5.16, p < 0.001; *Asic1a*^*+/+*^: t(45.6) = −1.70, p = 0.096), resulting in increased genotype difference at older ages (genotype-by-age interaction: t(56.4) = 2.025, p = 0.048; estimated difference in mice trained at P17: 13.4%; estimated difference in mice trained at P83: 32.2%) (Supplementary Fig. 2).

To ensure animals of all age groups learned, we compared baseline freezing on training day to freezing during acquisition. For all ages freezing during acquisition significantly differed from baseline freezing indicating that all animals acquired fear memory (P17: *Asic1a*^*-/-*^ t(15.5) = 5.735, p < 0.001, *Asic1a*^*+/+*^: t(15.5) = 6.841, p < 0.001; P23: *Asic1a*^*-/-*^ t(18.6) = 9.777, p < 0.001, *Asic1a*^*+/+*^: t(5.8) = 16.603, p < 0.001; P29: *Asic1a*^*-/-*^ t(4.4) = 9.063, p = 0.001, *Asic1a*^*+/+*^: t(6.2) = 16.707, p < 0.001; P42: *Asic1a*^*-/-*^ t(3.7) = 5.172, p = 0.008, *Asic1a*^*+/+*^: t(5.7) = 8.686, p < 0.001; P83: *Asic1a*^*-/-*^ t(7.6) = 5.928, p < 0.001, *Asic1a*^*+/+*^: t(3.67) = 19.544, p < 0.001).

### 3.3 ASIC1A disruption increases shock reactivity in adult females

As fear memory acquisition was impaired in *Asic1a*^*-/-*^ mice, we were wondering if this acquisition deficit is due to reduced ability to feel the foot shock. To probe this possibility, we evaluated shock reactivity at each developmental stage at which we performed fear conditioning, using motion in response to the first foot shock. At P17, there was a trend towards increased motion in *Asic1a*^*-/-*^ mice, but there was no significant genotype effect was observed in mice trained from P17 to P42 (P17: t = (27.5)= 1.909, p = 0.07; P23: t(10.2) = 0.307, p = 0.765; P29: t(5.7) = −0.518, p = 0.624; P42: t(2.6) = −0.818, p = 0.481) (Supplementary Fig. 3A-D). In mice trained at P83, we saw a significant genotype-by-sex interaction (t(28.8) = 2.509, p = 0.018), reflecting a higher shock reactivity in female, but not male, *Asic1a*^*-/-*^ mice compared to their *Asic1a*^*+/+*^ counterparts (females: t(9.3) = 2.915, p = 0.017, males: t = (8.4)= 0.589, p = 0.571) (Supplementary Fig. 3E, F). Due to these results, we performed sex-specific analysis on the effect of training age on shock reactivity. For male mice, our model showed increased shock reactivity with increasing age (t(30.6) = 6.662, p < 0.001), but no genotype-by-age interaction (t(40.3) = −1.382, p = 0.175), indicating that both genotypes had a similar increase in shock reactivity. For female mice, our model also showed increased shock reactivity with increasing age (t(38.8) = 8.579, p < 0.001), but in contrast to our result for males, we saw a genotype-by-age interaction (t(45.1) = −3.573, p < 0.001). This increasing shock reactivity might result in higher freezing during fear memory acquisition, and thus decrease genotype difference during acquisition.

### 3.4 In mice trained post-weaning, loss of ASIC1A impairs cue-evoked fear memory at 24 h after training

**Fig. 2:**
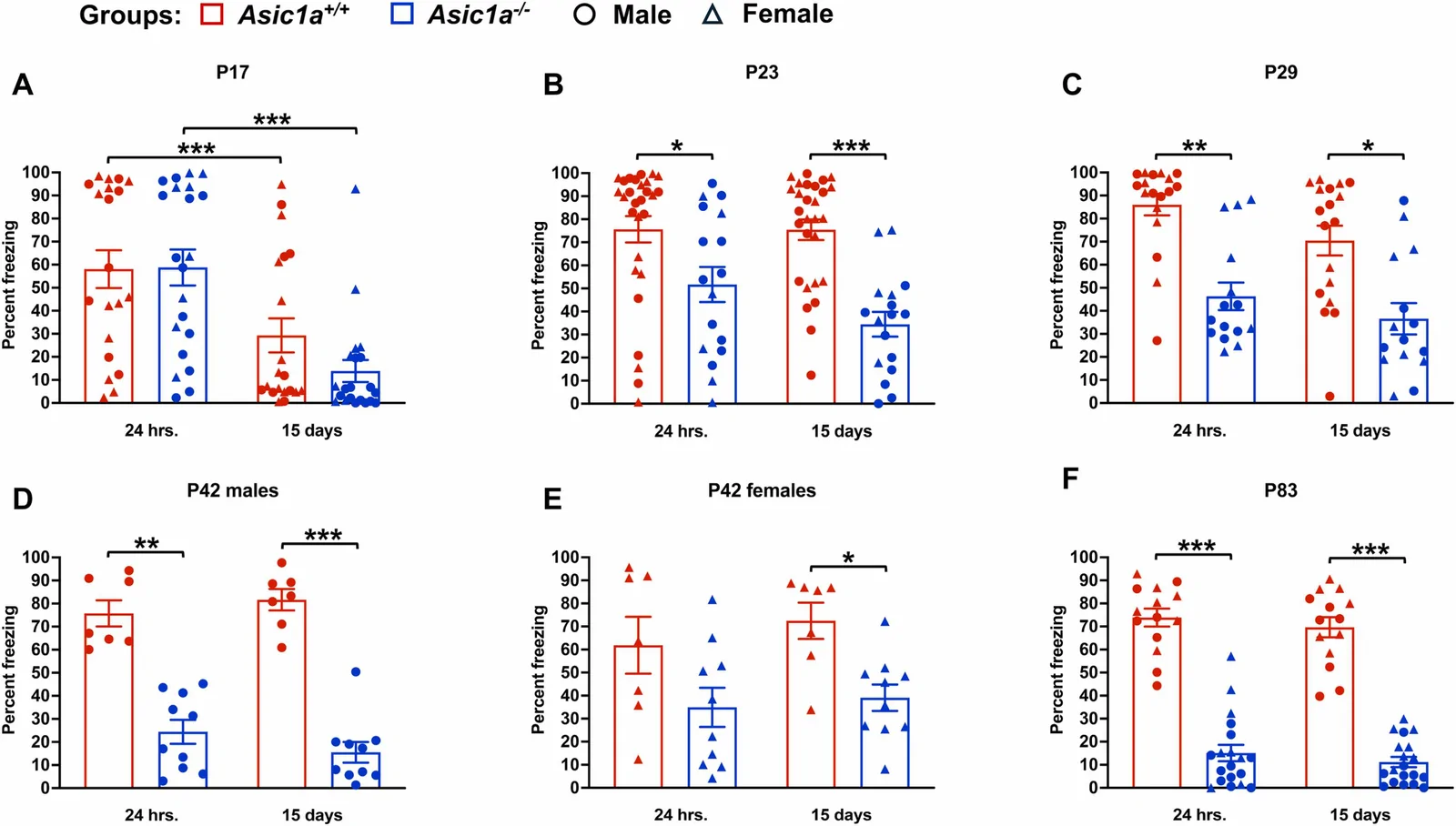
Age-specific effects of *Asic1a* disruption on cue-evoked fear memory. Cue-evoked freezing in *Asic1a* knockout (^-/-^, blue) vs. wild-type (^+/+^, red) mice measured at 24 h (hrs.) and 15 days post-training for mice trained at: P17 (A); P23 (B); P29 (C); P42, reported by sex: P42 males (D) and P42 females (E); and P83 (F). For mice trained at P42, males and females are shown in different graphs to highlight a significant sex-by-genotype interaction at 15 days after training. No sex-by-genotype interaction was detected for 24hrs after training. For 24hrs. after training, the respective male-specific p-value (WT m vs. KO m) is 0.002, and the respective female-specific p-value (WT f vs. KO f) is 0.072. Mouse numbers per experiment and sex distribution are the same as reported in Fig. 1.*p < 0.05, **p < 0.005, ***p < 0.001.

Despite the deficit in fear memory acquisition that we saw in *Asic1a*^*-/-*^ mice trained at P17, both genotypes froze similarly during cue presentation at 24 h after training (t(32.9) = −0.099, p = 0.922) (Fig. 2A, Table 1). In contrast, *Asic1a*^*-/-*^ mice trained at P23 and beyond froze less than *Asic1a*^*+/+*^ mice during cue presentation at 24 h after training (P23: t(18.1) = 2.568 p = 0.019; P29: t(6.5) = 4.562, p = 0.003; P42: t(5.8) = 4.080, p = 0.007; P83: t(6.3) = 9.261, p < 0.001) (Fig. 2B-F, Table 1). For mice trained at P83 we also saw a significant sex-effect, with females freezing more than males (t(29.6) = −2.224, p = 0.034). Freezing during cue presentation 24 h after training declined with age in *Asic1a*^*-/-*^ mice (t(64.9) = −4.653, p < 0.001), but not in *Asic1a*^*+/+*^ controls (t(41.7) = 0.762, p = 0.450), a difference that resulted in a significant genotype-by-age interaction: t(67.0) = 3.714, p < 0.001). While *Asic1a*^*-/-*^ mice trained at P17 froze similarly as *Asic1a*^*+/+*^ mice (estimated difference 1.1%), *Asic1a*^*-/-*^ mice trained at P83 froze dramatically less (estimated difference 59.2%) (Supplementary Fig. 4). Data on pre-cue freezing can be found in Supplementary Table 3.

### 3.5 In mice trained post-weaning, ASIC1A disruption impairs cue-evoked fear memory at 15 days after training

*Asic1a*^*-/-*^ and *Asic1a*^*+/+*^ mice were also tested for cue-evoked fear memory recall at 15 days after training (D15). In mice trained at P17, we saw no genotype effect at D15 (t(33.7) = 1.500, p = 0.143), which is in line with our result at 24 h after training (Fig. 2A, Table 1). In contrast, we did see a genotype effect for older mice: *Asic1a*^*-/-*^ mice trained at P23, at P29, or P83 froze less upon cue presentation on D15 than *Asic1a*^*+/+*^ mice (P23: t(42.0) = 5.661, p < 0.001; P29: t(6.5) = 3.040, p = 0.021; P83: t(30.0) = 14.658, p < 0.001) (Fig. 2B, C, F, Table 1). This genotype effect paralleled the results at 24 h after training. For mice trained at P23 or at P83 we saw a sex effect, as females froze more than males (P23: t(42.0) = −2.265, p = 0.029; P83: t(30.0) = −3.430, p = 0.002). For mice trained at P42, we saw a significant genotype effect in both sexes (males: t(11.2) = −7.057, p < 0.001; females: t(10.4) = −3.562, p = 0.005) (Fig. 2D, E, Table 1) and there was a significant sex-by-genotype interaction (t(29.8) = 2.780, p = 0.009), indicating that the genotype effect was larger in males than in females. In light of the sex-by-genotype interaction seen in mice trained at P42, we evaluated the effects of training age on cue-evoked fear memory at 15 days after training separately for each sex. For females we saw a significant genotype-by-age interaction (t(39.1) = 2.342, p = 0.024) while males exhibited a trend towards a genotype-by-age interaction (t(49.4) = 1.730; p = 0.090) (Supplementary Fig. 5A-B). Data on pre-cue freezing can be found in Supplementary Table 3.

When we were assessing the stability of cue-evoked fear memory in mice trained at P17, we saw a significant decline of cue-evoked freezing from 24 h to 15 days after training in both genotypes (*Asic1a*^*+/+*^ mice: t(27.6) = −4.082, p < 0.001; *Asic1a*^*-/-*^ mice: t(21.0) = −6.026, p < 0.001) (Fig. 2A, Table 1) with no significant difference in decline between genotypes (t(33) = −1.405; p = 0.169). In mice trained at P23, P29 or P83, within-genotype freezing at those timepoints remained stable (P23 *Asic1a*^*+/+*^ mice: t(8.9) = 0.049, p = 0.962; P23 *Asic1a*^*-/-*^ mice: t(17.8) = −1.033, p = 0.315; P29 *Asic1a*^*+/+*^ mice: t(6.4) = −1.946, p = 0.097; P29 *Asic1a*^*-/-*^ mice: t(5.8) = −1.197, p = 0.278; P83 *Asic1a*^*+/+*^ mice: t(4.91) = −0.915, p = 0.402: P83 *Asic1a*^*-/-*^ mice: t(7.38) = −0.676, p = 0.520) (Fig. 2B, C, F, Table 1), and there was also no difference in the stability of cue-evoked freezing between genotypes (P23: t(13.8) = −0.784, p = 0.447; P29: t(6.1) = 0.379, p = 0.718; P83: t(5.7) = 0.298, p = 0.776). Despite of the sex-by-genotype interaction we saw for freezing at 15 days after training in mice trained at P42, we saw no sex-by-genotype interaction for the stability of cue-evoked freezing between 24 h and 15 days after training. There was no significant change in freezing between genotypes between 24 h and 15 days after training (t(5.1) = −0.727, p = 0.500) and within-genotype freezing between those timepoints also remained stable (*Asic1a*^*+/+*^ mice: t(6.05) = 0.679, p = 0.522; *Asic1a*^*-/-*^ mice: t(4.14) = −0.330, p = 0.758). Due to the sex-by-genotype interaction at D15, freezing at 24 h and D15 are plotted by sex for mice trained at P42 (Fig. 2D, E).

### 3.6 Loss of ASIC1A has age-and sex-specific effects on context-evoked freezing tested at 48 h after training

**Fig. 3:**
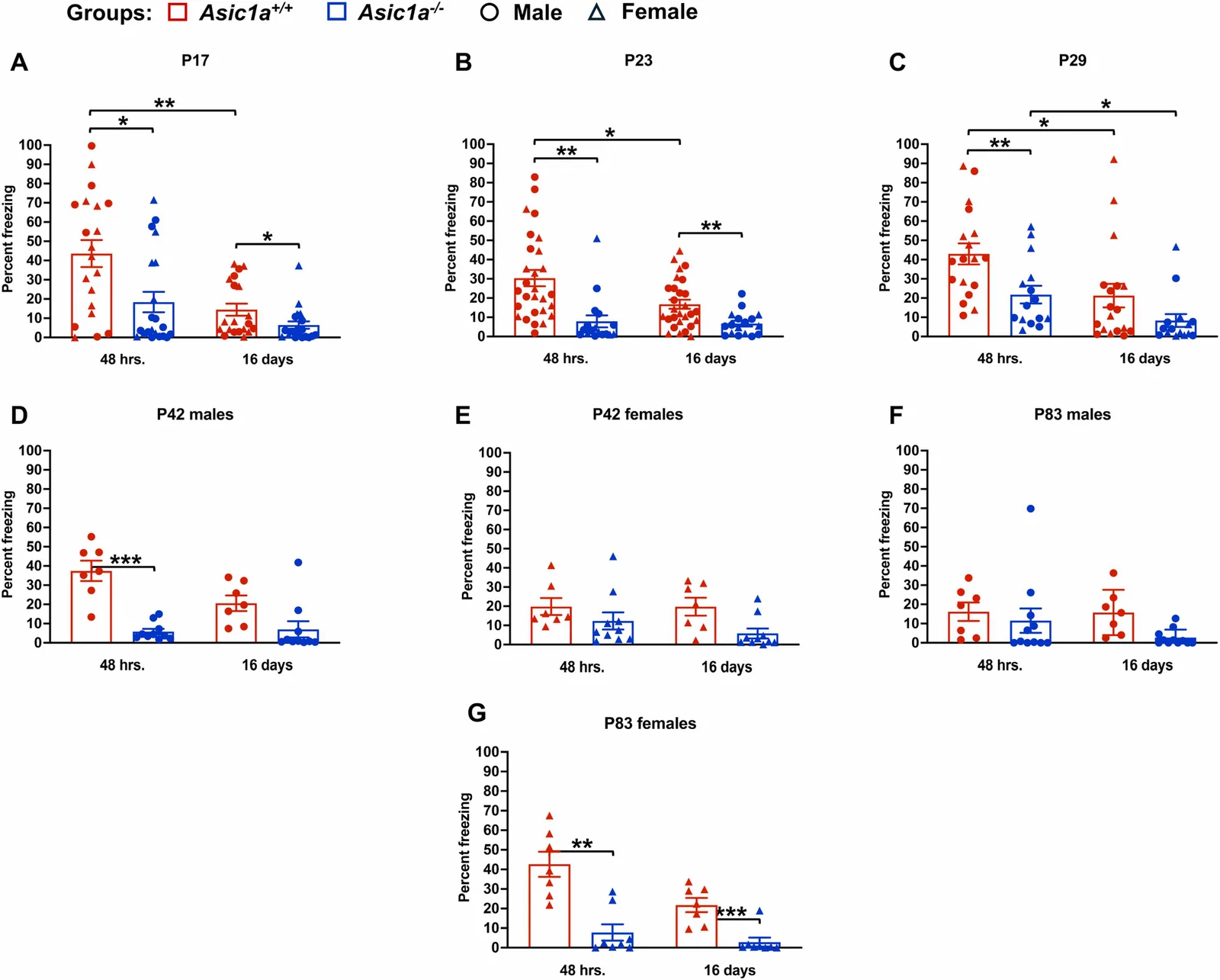
Age-specific effects of *Asic1a* disruption on contextual fear memory. Context-evoked freezing in *Asic1a* knockout (^-/-^, blue) vs. wild-type (^+/+^, red) mice at 48 h (hrs.) and 16 days post-training for mice trained at: P17 (A); P23 (B); P29 (C); P42, by sex: P42 males (D) and P42 females (E); and P83, by sex: P83 males (F) and P83 females (G). For mice trained at P42 and at P83, males and females are shown in different graphs due to significant sex-by-genotype interactions at 48 h after training. There were no sex-by- genotype interactions 16 days after training. For P42, the respective male-specific p-value (WT m vs. KO m) is 0.002 and the respective female-specific p-value (WT f vs. KO f) is 0.072. For P83 the respective male-specific p-value (WT m vs. KO m) is 0.002 and the respective female-specific p-value (WT f vs. KO f) is < 0.001. Mouse numbers per experiment and sex distribution are the same as reported in Fig. 1. *p < 0.05, **p < 0.005, ***p < 0.001.

Context-evoked freezing, tested at 48 h after training, was significantly lower in *Asic1a*^*-/-*^mice trained from P17 to P29 (P17: t(32.7) = 2.793, p = 0.009; P23: t(16.63) = 3.894, p = 0.001; P29: t(30.0) = 3.481, p = 0.002) (Fig. 3A-C, Table 1). In mice trained at P42, we saw a sex-by-genotype interaction (t(29.8) = 2.834, p = 0.008) indicating, lower freezing in *Asic1a*^*-/-*^ males (t(11.39) = −5.192, p < 0.001), but not in *Asic1a*^*-/-*^females (t(10.57) = −1.58, p = 0.144) compared to same-sex wildtypes (Fig. 3D, E, Table 1). In mice trained at P83, we also saw a significant sex-by-genotype interaction (t(28.3) = −2.118, p = 0.043) indicating a genotype effect in females, but not in males (males: t(8.9) = −1.057, p = 0.318, females: t(9.8) = −3.232, p = 0.009) (Fig. 3F, G, Table 1). Due to the sex-by-genotype interaction seen in mice trained at P42 and at P83, we evaluated the effects of training age on context-evoked fear memory separately for each sex. Neither for males nor for females did we see a genotype-by-age interaction (males: t(53.9) = −1.139, p = 0.260; females: t(37.3) = 1.541, p = 0.132) (Supplementary Fig. 6A-B).

Given the small difference between baseline freezing at day zero and freezing upon re-exposure to the training context at 48 h after training (Table 1) we performed follow up-analysis to probe if mice of each age group, genotype and sex retained memory about the training context by comparing baseline freezing at training day with context freezing at 48 h after training. This analysis revealed that in mice trained at P17 only *Asic1a*^*+/+*^ females showed a significant difference between baseline freezing at day zero and contextual fear memory recall at 48 h after training (t(37.8) = −3.29, p = 0.002), whereas a trend in this direction was observed in *Asic1a*^*+/+*^ males and *Asic1a*^*-/-*^ females (*Asic1a*^*+/+*^ males: t(44.0) = −1.92, p = 0.062; *Asic1a*^*-/-*^ females: t(44.1) = −1.79, p = 0.080) and no statistical difference was observed in *Asic1a*^*-/-*^ males (t(36.5) = 0.15, p = 0.882). At all other ages mice of both sexes and genotypes showed freezing above baseline levels when tested 48 h after training, indicating that they retained contextual memory (Supplementary Table 4).

### 3.7 Loss of ASIC1A has age-specific effects on contextual fear memory tested at 16 days after training

*Asic1a*^*-/-*^ and *Asic1a*^*+/+*^ mice were tested for the stability of contextual fear memory at 16 days post-training (D16). *Asic1a*^*-/-*^ mice trained at P17, P23 or P83 exhibited reduced context-evoked freezing when tested 16 days after training (P17: t(31.7) = 2.185, p = 0.036; P23: (t(42.0) = 3.095, p = 0.003; P83: t(30.0) = 6.415, p < 0.001) (Fig. 3A, B, F, G, Table 1). However, *Asic1a*^*-/-*^ mice trained at P29 showed no significant difference in freezing compared to age-matched *Asic1a*^*+/+*^ mice at 16 days after training (t(6.5) = 1.721, p = 0.132) (Fig. 3C, Table 1), which contrasts with the reduced, context-evoked freezing this age group showed at 48 h after training. *Asic1a*^*-/-*^ mice trained at P42 showed a strong trend towards lower freezing compared to *Asic1a*^*+/+*^ mice trained at the same age (t(6.3) = 2.414, p = 0.051) (Fig. 3D, E, Table 1). We saw a trend for lower freezing with increasing age at training (t(107.5) = −1.697, p = 0.093) and a significant interaction between age at training and genotype (t(60.1) = 2.13, p = 0.037), indicating lower freezing in *Asic1a*^*-/-*^ mice with increasing age at training upon re-exposure to the training context at 16 days after training (D16) (Supplementary Fig. 7).

In *Asic1a*^*+/+*^ mice trained at P17, context-evoked freezing declined significantly between 48 h and 16 days after training (t(19.9) = −3.795, p = 0.001) (Fig. 3A). *Asic1a*^*-/-*^ mice trained at P17 trended in the same direction (t(18.7) = −1.883, p = 0.075). Further analysis showed no genotype effect on the extent of this decline (t(30.3) = 1.593, p = 0.122). In mice trained at P23, context-evoked freezing between 48 h and 16 days after training significantly declined in *Asic1a*^*+/+*^ mice (t(6.37) = −2.759, p = 0.031) (Fig. 3B) and we saw a trend for a decline of context-evoked freezing in *Asic1a*^*-/-*^ mice (t(18.4) = −0.059, p = 0.095). There was also a trend for a genotype effect on the extent of decline of context-evoked freezing (t(12) = 1.802, p = 0.097) between 48 h and 16 days after training. In mice trained at P29, context-evoked freezing between 48 h and 16 days after training declined significantly in both genotypes (*Asic1a*^*+/+*^: t(6.5) = −3.390, p = 0.013; *Asic1a*^*-/-*^: t(6.4) = −2.602, p = 0.038) (Fig. 3C), with no genotype difference in the extent of decline (t(6.5) = 0.280, p = 0.788). In mice trained at P42, we saw a trend towards a genotype-by-sex interaction for the change in context-evoked freezing between 48 h and 16 days after training (t(29.8) = −1.797, p = 0.083). Follow-up analysis on the stability of context-evoked freezing in each sex did not reveal a genotype effect (males: t(11.3) = 1.216, p = 0.249; females: t(10.5) = −1.036, p = 0.323) (Fig. 3D, E). Given this lack of effect, we also assessed both sexes together and found no significant change in freezing between genotypes between 48 h and 16 days after training (t(4.5) = 0.120, p = 0.910) and within-genotype freezing between those timepoints also remained stable (*Asic1a*^*+/+*^ mice: t(5.45) = −1.453, p = 0.201); *Asic1a*^*-/-*^ mice t(3.52) = −1.548, p = 0.201). In mice trained at P83, though there was a sex-by-genotype interaction we saw for freezing at 48 h after training, we found no sex-by-genotype interaction for the stability of context-evoked freezing between 48 h and 16 days after training. There was no significant change in freezing between 48 h and 16 days after training in *Asic1a*^*+/+*^ versus *Asic1a*^*-/-*^ mice (t(4.9) = −0.226, p = 0.803) and within-genotype freezing between those timepoints also remained stable (*Asic1a*^*+/+*^ mice: t(3.67) = −1.427, p = 0.233); *Asic1a*^*-/-*^ mice t(7.58) = −1.989, p = 0.084). Due to the sex-by-genotype interaction at 48 h, freezing at 48 h. and D16 are visualized separately for each sex for mice trained at P42 and at P83.

## 4 Discussion

These data provide the first evidence that loss of ASIC1A has age-dependent effects on acquisition and recall of both cued and contextual fear memory. Our key findings are that: 1) Freezing during acquisition is lower in *Asic1a*^*-/-*^ mice across all ages tested, 2) Cued fear memory tested at 24 h and 15 days after training (D15) is impaired in post-weaning *Asic1a*^*-/-*^ mice, but is not impaired in P17 *Asic1a*^*-/-*^ mice, and 3) Context fear memory is largely impaired in *Asic1a*^*-/-*^ mice when tested at 48 h and at 16 days after training (D16). Interestingly, effects of ASIC1A disruption on freezing increased with age.

Fear memory acquisition in *Asic1a*^*-/-*^ mice was increasingly impaired with age, suggesting a developmental role of ASIC1A in fear learning. This idea is consistent with reports that ASIC1A is expressed very early in development, as early as embryonic day 12 (Alvarez de la Rosa et al. 2003). The BLA and hippocampus, key sites for fear learning and fear memory recall, robustly express ASIC1A (Wemmie et al., 2003, Coryell et al., 2007, Zha et al., 2006). It largely remains unclear if ASIC1A expression in these sites is developmentally regulated, though one paper reported a doubling in ASIC1A membrane expression in the hippocampus between the second and 12th postnatal week (Ma et al. 2019). If ASIC1A levels do increase in these areas in the timeframe assessed here, it could explain the progressively greater impairments in fear memory acquisition and cued fear memory recall in *Asic1a*^*-/-*^ mice. While we did not assess ASIC1A protein levels in this study, it would be an important that future studies look at the developmental time course of ASIC1A expression in the BLA and hippocampus to see if ASIC1A protein expression throughout development parallels the behavioral effects we described here.

These age-dependent deficits may also reflect changes in glutamate receptors across development. ASIC1A has been implicated in synaptic plasticity, with ASIC1A disruption impairing LTP in both the amygdala and hippocampus (Du et al., 2014; Chiang et al. 2015; Wemmie et al. 2002). Glutamate receptors are essential for LTP generation and fear conditioning (Maren, 1999, Maren, 2005). Two receptor subunits that have been critically implicated in fear memory acquisition, NR2B (Zhao et al., 2005, Zhang et al., 2008, Rodrigues et al., 2001) and GluA1 (Humeau et al., 2007, Feyder et al., 2007, Rumpel et al., 2005), are both developmentally regulated in the BLA. NR2B expression in the BLA decreases by almost half between P17 and adulthood, whereas GluA1 phosphorylation at Ser831, which is key for short-term memory formation, decreases by more than 80% in the same time period (Bessieres et al. 2019). Interestingly, ASIC1A disruption has been reported to alter expression and function of these glutamate receptor subunits. *Asic1a*^*-/-*^ mice have been reported to have decreased postsynaptic NR2B expression, and decreased NMDA receptor activity in striatal neurons (Ma et al., 2019, Yu et al., 2018). Moreover, altered GluA1 membrane trafficking, GluA1 phosphorylation and GluA1 protein expression have been reported in *Asic1a*^*-/-*^ mice (Li et al., 2019, Mango et al., 2017, Yu et al., 2018). Although these effects of ASIC1A disruption on glutamate receptors have not yet been evaluated in the fear circuit, they suggest a plausible mechanism for the age-dependent effects we observed. At younger ages, higher levels of NR2B expression and GluA1 Ser831 phosphorylation may partially compensate for the loss of ASIC1A, and this compensation may diminish as levels of these subunits decline.

Despite their impaired acquisition, *Asic1a*^*-/-*^ mice trained at P17 showed unimpaired cued fear memory recall when tested at 24 h and at 15 days after training (D15). This suggests the presence of a compensatory mechanism after acquisition that facilitates cued fear memory consolidation, leading to normal cued fear memory recall. This age-specific mechanism appears to be specific to cue-evoked fear memory, as contextual fear memory recall is impaired in *Asic1a*^*-/-*^ mice trained at P17, suggesting separable underlying processes.

A possible site where compensation might occur, is the BLA. Stabilization of new dendritic spines in the amygdala is essential for memory consolidation (Holtmaat et al., 2008, Muñoz-Cuevas et al., 2013). Multiple studies have implicated ASIC1A in spine dynamics (Zha et al., 2006, Kreple et al., 2014). Notably, there seem to be region-specific effects, with ASIC1A in the nucleus accumbens thought to oppose the formation of thin and stubby spines (Kreple et al. 2014), whereas increasing ASIC1A expression in the hippocampus increased dendritic spine density (Zha et al. 2006). Importantly, the role of ASIC1A in dendritic spine dynamics in the amygdala has not yet been assessed, though it is possible that altered spine dynamics in *Asic1a*^*-/-*^ mice might contribute to a compensatory mechanism after acquisition. If there were increased formation of dendritic spines or decreased turnover in the amygdala of *Asic1a*^*-/-*^ mice trained at P17, this could explain the milder impairment of cued fear memory acquisition and enhance consolidation. If this mechanism is age-dependent, and either absent or diminished in mice trained at older ages, it would explain the deficit we see in cued fear memory recall at 24 h after training at all older ages.

Cue-evoked freezing in mice trained at P17 declined significantly between 24 h and 15 days after training (D15), regardless of genotype, but remained stable in mice trained at all older ages. This might reflect developmental differences in synaptic pruning. Spine turnover is high at P17 (Holtmaat et al., 2005, Runge et al., 2020), and the preservation of spines plays an important role for memory maintenance after initial consolidation (Basu and Lamprecht, 2018). Though direct comparisons in spine stability between P17 and P23 are not available, there is a general trend of increasing spine stability with age, raising the possibility that low spine stability at P17 contributes to the decline in cue-evoked freezing.

In contrast to cued fear memory recall, contextual fear memory recall at 48 h was largely impaired in *Asic1a*^*-/-*^ mice at all ages. Follow-up analysis showed that male *Asic1a*^*-/-*^ mice trained at P17 showed no evidence for retention of contextual fear memory at 48 h after training. However, female *Asic1a*^*+/+*^ mice showed significant retention of contextual fear memory after training, and female *Asic1a*^*-/-*^ mice and male *Asic1a*^*+/+*^ mice trended in the same direction. At all older ages, both sexes and genotypes retained contextual fear memory at 48 h after training. It has been reported that Pavlovian fear conditioning before P17 does not to induce stable context-evoked freezing in mice when tested 24 h after training and the hippocampus is essential for the development and retention of contextual fear memory (Akers et al. 2012). Our results might indicate that hippocampal development necessary to retain contextual fear memory might be delayed in *Asic1a*^*-/-*^ males, resulting in a lack of retention of contextual fear memory in these mice at 48 h after training. Our results in mice trained at P17 also suggest a developmental dissociation between the retention of context-evoked and cue-evoked freezing. Interestingly, a similar developmental dissociation between cued and contextual fear memory recall has been reported for rats (Rudy, 1993, Rudy and Morledge, 1994).

Contrasting the stability of cue-evoked freezing in all mice trained post-weaning, context evoked fear memory tended to decline between 48 h and 16 days after training in all mice trained before P42. Regional differences in maturation of the circuits for cued and contextual fear memory could possibly explain this developmentally later onset of stability of context-evoked freezing – relative to cue-evoked freezing. In line with this a delayed onset of context evoked fear memory – relative to cue evoked fear memory – has been reported for rats (Burman et al. 2014). While no study tested parameters identical to ours, it has been found that retention of 30-day memory for both, cue and context, emerges at P25 (Samifanni et al. 2021). One possible explanation for the relative instability of contextual fear memory in younger mice could be the greater levels of pruning seen at younger ages and the differences in stability between cued and contextual fear memory could reflect differences in pruning in their respective underlying circuits (Holtmaat et al., 2005, Runge et al., 2020, Koss et al., 2014, Parato et al., 2019).

Studying sex-effects was not the primary objective of this study, and thus experimental groups were not necessarily powered to study this. Despite this, we did observe some sex-dependent effects. It is interesting that sex-genotype interactions for cued and contextual fear memory emerged in mice trained at P42, which may reflect hormonal changes occurring as mice reach sexual maturity around this time (Bell, 2018). While sex differences in fear conditioning have been reported, they are complex, and the reported results are equivocal (Bauer, 2023). So far sex differences in ASIC1A expression have not yet been studied.

## 5 Conclusion

Our study provides the first evidence of age-dependent effects of ASIC1A on Pavlovian fear conditioning and provides the first evidence that its effects on cued and contextual fear conditioning are separable. It is remarkable that effects of ASIC1A deficiency on cue-evoked fear memory recall emerge in a timeframe that comprises the late preweaning period (P18) and the early postweaning period (P23). This is the time when the appropriate assessment of threats becomes vital. Context-evoked fear memory, however, is largely impaired in *Asic1a*^*-/-*^ mice of all ages. *Asic1a*^*-/-*^ mice trained at P17 do not robustly acquire contextual fear memory, but *Asic1a*^*-/-*^ mice trained at older ages acquire context memory, suggesting a delayed circuit development in those mice. Notably, the stability of context-evoked fear memory over time is achieved at older ages than stability of cue-evoked fear memory, also suggesting a delayed development of the circuits relevant for contextual fear memory.

Overall, our results suggest a complex role of ASIC1A in the maturation and/or function of circuits relevant to Pavlovian fear conditioning.

## CRediT authorship contribution statement

**R.J. Taugher-Hebl:** Writing – review & editing. **A. Berns:** Investigation. **M. Jones:** Investigation. **A. Townsend:** Investigation. **A. Eagen:** Investigation. **Langbehn DR:** Software, Formal analysis. **H. Janouschek MD:** Writing – review & editing, Visualization, Formal analysis.

## Declaration of Competing Interest

The authors declare that they have no known competing financial interests or personal relationships that could have appeared to influence the work reported in this paper.
