# Supplementary material for: The effects of acid-sensing ion channel-1A on conditioned fear memory are age-dependent
Source: IBRO Neurosci Rep. 2026 Aug 5;21:595–603. doi: 10.1016/j.ibneur.2026.08.002 (PMC13499391; doi:10.1016/j.ibneur.2026.08.002)
Supplement: Supplementary file 1 [file mmc1.docx]

**Supplementary Figure 1: Timeline for fear conditioning experiments.**

**A)** Contexts used for training and assessment of cue-evoked and contextual fear memory recall and maintenance. Musical note = tone presentation, i.e., cue; red lightning bolts= foot shocks; gray floor (grid) and bleach = context A; white floor, triangular roof, peppermint odor (green leaves) = context B. **B)** Timelines for training and measurement of memory. Cue test on D15 will be also referred to as cued fear memory maintenance and D16 will be referred to at contextual fear memory maintenance.

**Supplementary Figure 2:** **The effect of ASIC1A disruption on fear memory acquisition increases with age.**

N for P17: 20 wild-type (8 m, 12 f) and 21 knockout (13 m, 8 f) mice. N for P 23: 28 wild-type (14 m, 14 f) and 17 knockout (11 m, 6 f) mice. N for P29: 18 wild-type (10 m, 8 f) and 15 knockout (7 m, 8 f) mice. N for P42: 14 wild-type (7 m, 7 f) and 20 knockout (10 m, 10 f) mice. N for P83: 14 wild-type (7 m, 7 f) and 19 knockout (11 m, 8 female) mice.

**Supplementary Figure 3: At P83, female *Asic1a* knockout mice showed more motion in response to the first foot shock with no genotype effect in all other comparisons.**

Maximum motion index for knockout (^-/-^, blue) and wild-type (^+/+^, red) mice trained at: P17 **(A)**; P23 **(B)**; P29 **(C);** P42 (**D)**; and P83 as reported by sex: (**E)** P83 females and (**F)** P83 males. For P17 – P42 both sexes are pooled, since there was no genotype-by-sex interaction. For P83, females and males are shown separately because there was a genotype-by-sex interaction indicating that female knockout mice had increased shock reactivity relative to female wild-type mice. Mouse numbers per experiment and sex distribution are the same as reported in Supplementary Figure 2. *p<0.05, **p<0.005, ***p<0.001

**Supplementary Figure 4: The effect of ASIC1A disruption on cue-evoked fear memory at 24 hours after training increase with age.**

N for P17: 20 wild-type (8 m, 12 f) and 21 knockout (13 m, 8 f) mice. N for P 23: 28 wild-type (14 m, 14 f) and 17 knockout (11 m, 6 f) mice. N for P29: 18 wild-type (10 m, 8 f) and 15 knockout (7 m, 8 f) mice. N for P42: 14 wild-type (7 m, 7 f) and 20 knockout (10 m, 10 f) mice. N for P83: 14 wild-type (7 m, 7 f) and 19 knockout (11 m, 8 female) mice.

**Supplementary Figure 5: In female mice, age and genotype determine freezing during cued fear memory recall at 15 days after training**

**A)** In male mice there was a trend towards an interaction between age-at-training and genotype. **B)** In female mice there was a significant interaction between age-at-training and genotype. Mouse numbers and sex distribution are the same as reported in Supplementary Figure 2.

**Supplementary Figure 6: There is no significant interaction between context-evoked freezing at 48 hrs. after training and training age**

There was no genotype-by-age interaction for context-evoked freezing at 48 hrs. after training in either in male **(A)** or female **(B)** mice.

Mouse numbers and sex distribution are the same as in Supplementary Figure 2.

**Supplementary Figure 7:**

There was no genotype-by-age interaction in male mice **(A)**, but female knockout mice froze less upon context re-exposure 16 days after training and this effect increased with age **(B)**. Mouse numbers and sex distribution are the same as in Supplementary Figure 2.

**Supplementary Table 1: Number of litters used**

| **Age at training** | **Number of litters used** | |
| --- | --- | --- |
|  | ***Asic1a^+/+^*** | ***Asic1a^-/-^*** |
| **P17** | 11 | |
| **P23** | 7 | 9 |
| **P29** | 5 | 4 |
| **P42** | 4 | 4 |
| **P83** | 3 | 6 |

**Supplementary Table 1: Number or litters used for the respective age groups.**

For pre-weaning (P17) fear-conditioning experiments littermate controls (*Asic1a^+/+^* and *Asic1a^-/-^*) were generated by *Asic1a^+/-^* x *Asic1a^+/-^* breeding, as mentioned in the methods section. For this age group litters were only used if they had at least one *Asic1a^+/+^* and one *Asic1^–/–^* mouse, therefore, the same litters were used for *Asic1a^+/+^* and *Asic1^–/–^* mice. For post-weaning fear conditioning experiments mice were generated by *Asic1a^–/–^* x *Asic1^–/–^ and Asic1a^+/+^*x *Asic1a^+/+^* breeding, therefore, *Asic1a^+/+^* and *Asic1^–/–^* mice came from different litters.

**Supplementary Table 2: Age-adjusted motion thresholds**

| **Training age** | **Training** | **Cue test 1** | **Context test 1** | **Cue test 2** | **Context test 2** |
| --- | --- | --- | --- | --- | --- |
| **P17** | 1 | 1 | 1 | 5 | 5 |
| **P23** | 1 | 1 | 1 | 10 | 10 |
| **P29** | 5 | 5 | 5 | 10 | 10 |
| **P42** | 10 | 10 | 10 | 10 | 10 |
| **P83** | 10 | 10 | 10 | 10 | 10 |

**Supplementary Table 2:**

Motion thresholds were optimized for mouse age based on hand scoring of a representative group of mice, as listed above and used for autoscoring using the VideoFreeze^TM^ software (Med Associates). Freeze duration was kept at 30 frames for mice in all age groups.

**Supplementary Table 3: Age-dependent effects of ASIC1A disruption on pre-cue freezing.**

| **Age at training** | **Pre-cue freezing 24 hrs. after training** | | | **Pre-cue freezing 15 days after training** | | |
| --- | --- | --- | --- | --- | --- | --- |
|  | ***Asic1a^+/+^*** | ***Asic1a^-/-^*** | **p** | ***Asic1a^+/+^*** | ***Asic1a^-/-^*** | **p** |
| **P17** | 20.5±26.0 | 12.4±13.8 | 0.180**^†^** | 1.3±2.2 | 0.5±1.3 | 0.083**^†^** |
| **P23** | 29.9±31.9 | 5.6±11.1 | 0.010***^†^** | 7.2±13.0 | 1.6±1.9 | 0.053**^†^** |
| **P29** | 29.4±30.7 | 11.2±18.8 | 0.147**^†^** | 9.7±14.4 | 3.1±5.4 | 0.037***^†^** |
| **P42** | 8.3±9.5 | 3.6±4.2 | 0.471**^†^** | M:  13.3±8.2  F:  7.3±6.0 | M:  0.6±0.7  F:  2.9±2.7 | M:  0.001***^†^**  F:  0.154**^†^** |
| **P83** | M:  6.1±4.9  F:  25.8±15.8 | M:  2.0±4.7  F:  2.6±4.3 | M:  0.063**^†^**  F:  0.001***^†^** | M:  6.9±11.2  F:  25.2±19.2 | M:  2.4±4.6  F:  0.9±1.1 | M:  0.325**^†^**  F:  0.001***^†^** |

**Supplementary Table 3: Comparison of pre-cue freezing between *Asic1a*^+/+^ *and Asic1a*^-/-^ mice across development 24 hrs. and 15 days after training.**

A higher degree of pre-cue freezing is consistent with a higher level of context generalization.

24 hours after training there were no sex-effects for animals trained P17 through P42. For animals trained at P83 there was a significant genotype-by-sex interaction (t(28.5)=-2.309, p=0.028).

15 days after training, we saw a sex effect in animals trained at P17 (t(32.9)=-2.887, p=0.007), but no genotype-by-sex interaction. For animals trained at P23 and P29 we saw no sex-effect and no genotype-by-sex interaction. For animals trained at P42 and P83 we saw a genotype-by-sex interaction (P42: t(28.2)=3.215, p= 0.003; P83: t(29.0)=-2.624, p=0.014).

Due to the sex-by-genotype interactions we saw, we evaluated the effects of training age on pre-cue freezing at 24 hrs. and 15 days after training separately for each sex. We only saw a significant genotype-by-age interaction for pre-cue freezing at 15 days after training (p<0.001), older female *Asic1a*^+/+^ mice froze more.

In cases of significant genotype-by-sex interaction data for males and females are listed separately, in all other cases data for both sexes is combined. Data are presented as means ± standard deviation from the raw data. P values are calculated by the mixed effect linear effect model controlling for sex as an additional fixed effect and litter as a random effect. *Statistically significant within the fitted linear model. **^†^** Based on square root transformations for improved statistical significance accuracy. *Statistically significant

**Supplementary Table 4: Comparison of baseline freezing during training and freezing during contextual fear memory recall at 48 hours after training**

|  | ***Asic1a^+/+^*** | | ***Asic1a^-/-^*** | |
| --- | --- | --- | --- | --- |
| **Age at training** | ***males*** | ***females*** | ***males*** | ***females*** |
| **P17** | t(44.0)=-1.92, p=0.062 | t(37.8)=-3.29, p=0.002* | t(36.5)=0.15, p=0.882 | t(44.1)=-1.79, p=0.080 |
| **P23** | t(51.5)=-7.68, p<0.001* | t(51.5)=-6.79, p<0.001* | t(51.5)=-2.23, p=0.030* | t(51.5)=-3.23, p=0.002* |
| **P29** | t(30.2)-9.23, p<0.001* | t(30.2)=-9.16, p<0.001* | t(30.2)=-5.14, p>0.001* | t(30.2)=-7.48, p<0.001* |
| **P42** | t(17.3)=-8.96, p<0.001* | t(20.2)=-6.56, p<0.001* | t(12.3)=-5.31, p<0.001* | t(17.5)=-4.49, p<0.001* |
| **P83** | t(30.2)=-4.22, p<0.001* | t(30.2)=-6.87, p<0.001* | t(30.2)=-3.25, p<0.001* | t(30.2)-2.47, p=0.019* |

**Supplementary Table 4:**

In mice trained at P17 only female *Asic1a^+/+^* mice show clear evidence for context learning, demonstrated by the significant difference between baseline freezing at training day and freezing during context re-exposure at 48 hours after training. Age-matched male *Asic1a^+/+^* and female *Asic1a^-/-^* mice show some evidence for context learning, which does only reach the level of a statistical trend, while age-matched male *Asic1a^-/-^* mice do not appear to learn the training context. Mice trained at all ages older than P17 show significant evidence for context learning independent of sex or genotype**.** *Statistically significant
